# Supplementary material for: Saccharomyces boulardii (CNCM I-745) improves intestinal damage in sepsis by remodeling intestinal flora structure
Source: Front Cell Infect Microbiol. 2025 Jul 31;15:1602792. doi: 10.3389/fcimb.2025.1602792 (PMC12350329; doi:10.3389/fcimb.2025.1602792)
Supplement: Supplementary file 1 [file DataSheet1.zip › summary/summary/7_Differential_analysis/3_Indicator_result/7_Species/Species_indicator_Group.pdf]

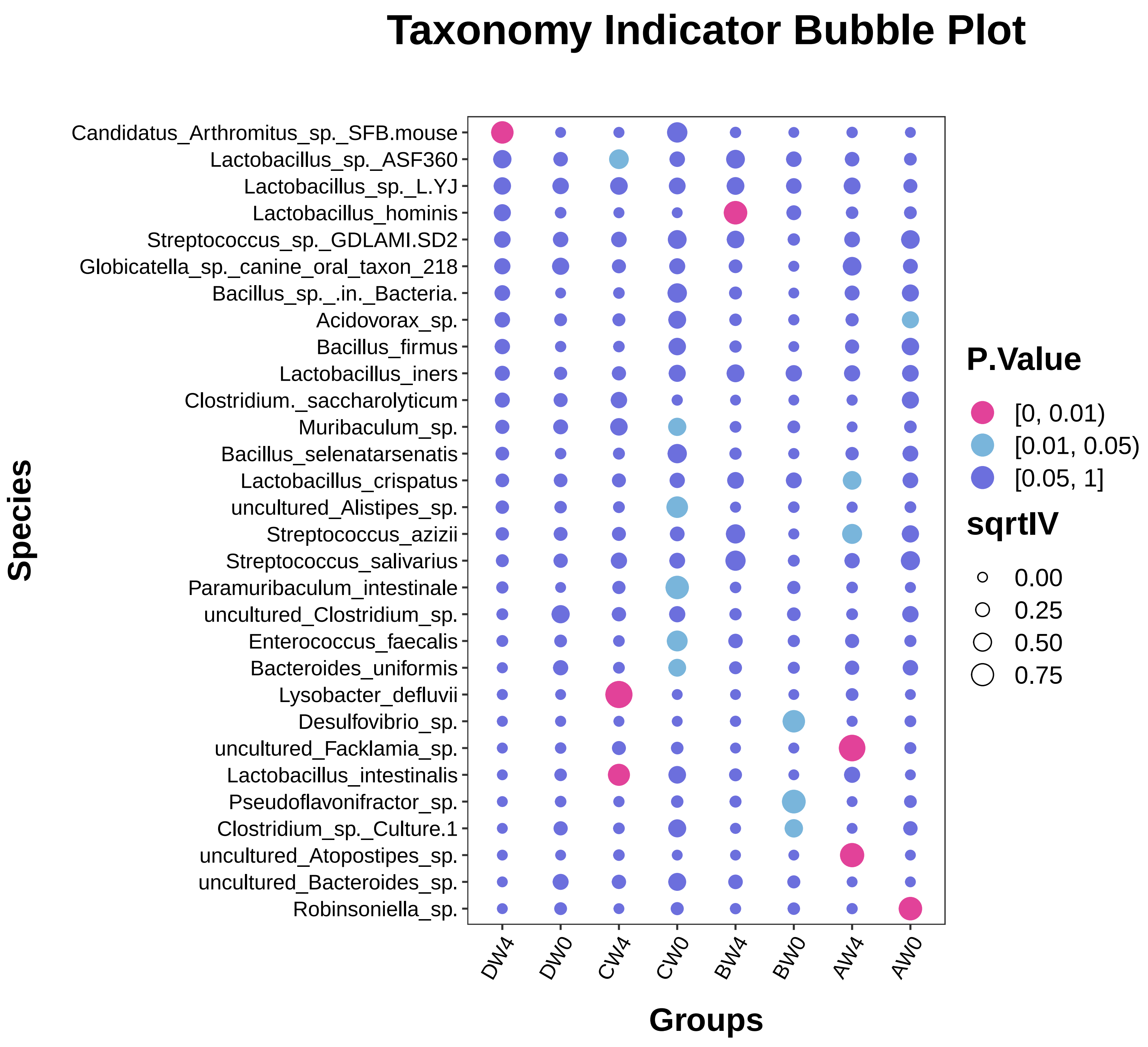
Taxonomy Indicator Bubble Plot
Species
Candidatus_Arthromitus_sp._SFB.mouse
Lactobacillus_sp._ASF360
Lactobacillus_sp._L.YJ
Lactobacillus_hominis
Streptococcus_sp._GDLAMI.SD2
Globicatella_sp._canine_oral_taxon_218
Bacillus_sp._.in._Bacteria.
Acidovorax_sp.
Bacillus_firmus
Lactobacillus_iners
Clostridium._saccharolyticum
Muribaculum_sp.
Bacillus_selenatarsenatis
Lactobacillus_crispatus
uncultured_Alistipes_sp.
Streptococcus_azizii
Streptococcus_salivarius
Paramuribaculum_intestinale
uncultured_Clostridium_sp.
Enterococcus_faecalis
Bacteroides_uniformis
Lysobacter_defluvii
Desulfovibrio_sp.
uncultured_Facklamia_sp.
Lactobacillus_intestinalis
Pseudoflavonifractor_sp.
Clostridium_sp._Culture.1
uncultured_Atopostipes_sp.
uncultured_Bacteroides_sp.
Robinsoniella_sp.
DW4
DW0
CW4
CW0
BW4
BW0
AW4
AW0
Groups
P.Value
[0, 0.01)
[0.01, 0.05)
[0.05, 1]
sqrtIV
0.00
0.25
0.50
0.75
